# Medical students’ positive perception towards vaccination is strongly correlated to protective diphtheria antibody after Td vaccination

**DOI:** 10.1016/j.bbih.2021.100362

**Published:** 2021-10-05

**Authors:** Emmy Hermiyanti Pranggono, Marita Restie Tiara, Tohari Catur Pamungkas, Esti Syafriati, Kuswandewi Mutyara, Rudi Wisaksana

**Affiliations:** a Department of Internal Medicine, Hasan Sadikin General Hospital/Universitas Padjadjaran, Jl. Pasteur No. 38 Bandung, West Java, Indonesia; b Faculty of Medicine, Universitas Padjadjaran, Jl. Eyckman No. 38 Bandung, West Java, Indonesia; c Department of Internal Medicine, Al-Ihsan General Hospital, Jl. Kiastramanggala Bale Endah, Bandung, West Java, Indonesia; d Department of Public Health, Faculty of Medicine, Universitas Padjadjaran, Jl. Eyckman No. 38, Bandung, West Java, Indonesia

**Keywords:** Psychoneuroimmunology, Diphtheria, Vaccination, Medical students, Perception, Stress, Antibody, Vaccine hesitancy

## Abstract

Negative perception towards vaccination is one of the reasons for low coverage of diphtheria immunization in Indonesia. Perception, which is difficult to change, is related to stress level, possibly influences outcome of diseases, and also vaccination. This study aims to identify the correlation between perception of diphtheria vaccination and antibody response after vaccination.

This study used secondary data from two unpublished studies on 30 medical interns in Hasan Sadikin Hospital, Bandung, West Java, after diphtheria outbreak, from June to July 2019. Antibody level after diphtheria emergency vaccination was measured using ELISA and perception towards vaccination was measured using a questionnaire. Perception towards vaccination was expressed as perception score and was divided into 4 components: perceived threat, benefit, barrier, and cues to action. Higher perception score indicated more positive perception towards vaccination. Diphtheria antibody level was grouped into reliable protection (≥0,10 IU/mL) or unreliable protection (<0,10 IU/mL). Statistical correlation analysis was done with GraphPad Prism version 7.0.

Most of our subjects were female. Median age was 22 (20–24) years old. Median time elapsed between vaccination date and measurement of antibody level was 18 (6–18) months. Median antibody level was 0,28 (0,09–3,47) IU/mL. Twenty-three subjects (82,1%) had reliable protection. Subjects with reliable protection had more positive perception compared to unreliable protection (perception score 80,6 ​± ​5,4 vs 69,0 ​± ​1,8, *p* ​= ​0,0001). Subjects with reliable protection had less perceived barrier for vaccination (15,6 ​± ​2,1 vs 13,0 ​± ​1,8, *p* ​= ​0,0083). Perception score showed strong, positive correlation to reliable protection against diphtheria (R ​= ​0,705, p ​< ​0,001). Perceived barrier and threat showed positive correlation to reliable protection (R ​= ​0,489, p ​= ​0,008 and R ​= ​0,402, p ​= ​0,034).

In conclusion, perception towards diphtheria vaccination is strongly correlated to protective antibody. Improving perception of vaccination are needed to overcome vaccine hesitancy.

## 1 Introduction

One of the reasons for diphtheria outbreak in 2017–2018 in Indonesia was poor coverage of diphtheria immunization (Tosepu et al., 2018). Diphtheria outbreaks occurred periodically in Indonesia. The 2017–2018 outbreak observed an exponential increase in the number of cases as well as death. This prompted the Government to launch outbreak response immunization (Tosepu et al., 2018). As health care workers were in the front lines of such situation, they were urged to get booster tetanus-diphtheria vaccination if they had not had one within the previous 10 years, as per national immunization guide (Indonesian Society of Internal Medicine, 2017).

Childhood immunization program had been unable to meet universal coverage of immunization targets despite Government efforts (Agency, 2019). Indonesia consisted of a vast geographical area and large population, which made logistical and administrative problems in vaccination more challenging. The country is also made up of myriad of cultures, beliefs, and religions, each with their own beliefs about vaccination (Pronyk et al., 2019). This created obstacle in convincing parents for vaccinating their children as parents were also influenced by religious leaders and cultural norms rather than healthcare providers only (Pronyk et al., 2019; Syiroj et al., 2019). Vaccine hesitancy was also found in health care workers in Indonesia, further compounding the problem (Harjaningrum et al., 2013). Vaccine hesitancy probably arose due to misperceptions about vaccination (Syiroj et al., 2019; Yufika et al., 2020; Harapan et al., 2018).

Perception develops since early age, influenced by society and family values (Benyamini and Friedman, 2011; Castro et al., 2012). As majority of Indonesians are Muslims, Islamic beliefs, passed on in families and by religious leaders, play important role in development of perception on vaccination. The belief that certain vaccines contain *haram* ingredients make some parents hesitant on giving the vaccine to their children (Syiroj et al., 2019). Norms also dictate some Indonesians to follow their small group leaders. For example, parents in a prayers' group (*pengajian*) will likely not vaccinate their children if their *ustadz* (leader in a prayers’ group) ask them not to. This contributed in forming the perception that vaccination must be harmful as their leader asks them not to do it (Syiroj et al., 2019).

The concept of illness perceptions was first introduced by Leventhal through the Common Sense Model of Self-Regulation (Leventhal et al., 2016). This model proposed that the way an individual perceived illness or somatic complaints were influenced by their cognitive and emotional representations of the illness (Hagger et al., 2017). Illness perceptions have been shown to influence stress level in patients with diseases, such as leukemia, gastrointestinal cancer, and vaccination (Westbrook et al., 2016; Miceli et al., 2019). Patients with perceptions of more severe disease, less effective treatments, and more negative disease-related quality of life have higher stress level than their counterparts (Benyamini and Friedman, 2011; Westbrook et al., 2016; Miceli et al., 2019). Stress has been shown to negatively impact disease occurrence and outcome (Lu et al., 2019; Adjei et al., 2019). Hence, it is in clinicians’ interest to study how to modify perceptions.

Stress has also been shown to negatively impact immunity (Slavich, 2019). Psychoneuroimmunology studies show that both acute and chronic stress might negatively impact the outcome of vaccination programs (Whittaker, 2018). Subjects with higher perceived stress have poorer antibody responses to influenza and Hepatitis B vaccination (Glaser et al., 1992; Kiecolt-Glaser et al., 1996). Subjects with more positive affect are also found to have better antibody response to vaccination (Marsland et al., 2006; Jenkins et al., 2018). These show the presence of mind-body connections between the brain and immune response. Indeed, stress has been shown to increase norepinephrine and cortisol levels, which affect the immune system (Slavich, 2019). Norepinephrine modulates the immune system by increasing transcription of pro-inflammatory cytokine genes, such as interleukin-1, interleukin-6, and transcription nuclear factor (Grebe et al., 2010). These inflammatory cytokines activate neutrophils, lymphocytes, and macrophages as well as stimulate the hypothalamic-pituitary-adrenal (HPA) axis to release cortisol. Inflammatory cytokines are also responsible in regulating fever, suppressing appetite, and increasing pain sensitivity (Grisanti et al., 2011). The increase in inflammatory cytokines also cause a decrease in antiviral activity, which are usually modulated by anti-inflammatory cytokines such as interleukin-4 and interleukin-10 (Slavich, 2019). Cortisol, released by activation of HPA axis, is one of the most potent immunomodulatory substances in the body. The hormone increases inflammatory activity in the short term, by rallying inflammatory cells to sites of injury and intensifying immunologic response to challenges (Sorrells et al., 2009). However, chronic exposure to high levels of cortisol leads to glucocorticoid resistance, which dampens the effect of cortisol to inflammatory cells, leading to increased inflammation despite reduced adaptive immune activity (Miller et al., 2002). Lastly, the brain has also been shown to be connected to the immune response via the meningeal lymphatic system in the dural sinuses. The meningeal lymphatic system drains to the deep cervical lymph nodes. This shows that there is a possibility of direct neuro-immunologic modulation (Slavich, 2019; Louveau et al., 2015).

As perceptions influence stress, and stress influences response to vaccination, the aim of this study is to determine the correlation between perception on diphtheria vaccination and antibody response.

## 2 Methods

### 2.1 Study design

This cross-sectional correlational study was conducted using secondary data from two previous, unpublished studies which separately measured perception towards diphtheria vaccination using questionnaire and diphtheria toxin antibody levels (Pamungkas et al., 2019; Syafriati et al., 2020). The study participants consisted of medical students in Hasan Sadikin General Hospital from June to July 2019. Most students got tetanus-diphtheria (Td) vaccination before their clinical rotations in addition to Hepatitis B vaccination due to the occurrence of diphtheria outbreak in 2017–2018.

In the previous studies, participants filled the questionnaire and had their blood samples drawn for measurement of initial diphtheria toxin antibody levels. The participants then were given emergency Td vaccination which was part of the Government's outbreak response immunization amidst earlier diphtheria outbreak. One month after the vaccination, their blood samples were again obtained to measure diphtheria toxin antibody levels after emergency vaccination (Pamungkas et al., 2019; Syafriati et al., 2020).

Inclusion criteria was participants of previous studies which had Td vaccination prior to undergoing clinical internships. Subjects with uncertain history of basic childhood vaccination were excluded. Twenty-eight out of the 30 participants of the previous studies satisfied the inclusion and exclusion criteria for this study. Despite the small sample size, power analysis for correlation study was satisfied for α ​= ​0,10, β ​= ​0,20 and assumed correlation size R ​= ​0,50, which yielded a minimum sample size of 23. Data on demography, perceptions of diphtheria vaccination, and initial diphtheria toxin antibody levels from the two previous studies were collected. Correlation analysis was performed between perception and presence of initial protective diphtheria toxin antibody.

This paper was written according to the Strengthening the Reporting of Observational studies in Epidemiology (STROBE) guideline.

### 2.2 The questionnaire

The questionnaire used was titled “Knowledge, Perception, and Practice of Diphtheria Booster Vaccination in Health Care Workers”, which was created by Infectious Disease Working Group, Faculty of Medicine, Universitas Padjadjaran in April 2019. The questionnaire consisted of 4 parts, which were: (1) sociodemographic questionnaire, (2) knowledge of diphtheria and diphtheria vaccination, (3) perception towards diphtheria vaccination, and (4) practice of diphtheria vaccination. It was validated in a group of 30 other medical students prior to use. The questionnaire showed good validity (p-value <0,05 using Pearson's correlation test) and good internal consistency for items in the perception scale (Cronbach alpha coefficient of 0,812). (Pamungkas et al., 2019).

The sociodemographic questionnaire consisted of variables that might influence perception towards diphtheria vaccination and the diphtheria toxin antibody levels, which are the following: (1) gender, (2) age, (3) time since last Td booster, (4) previous negative experience related to vaccination, (5) pocket money amount per month, (6) place of residence. The last two variables were not included in the analysis (Pamungkas et al., 2019).

The knowledge part of the questionnaire consisted of 10 statements on diphtheria symptoms, complications, vaccination schedules. The subjects responded with a true/false answer. A score of 1 is given for each correct response to give a total knowledge score between 0 and 10. The higher the knowledge score, the more knowledgeable the subject was (Whittaker, 2018).

The perception part of the questionnaire consisted of 19 statements. The statements were created based on the Health Belief Model, consisting of four components of the model which were: (1) perceived threat of diphtheria (4 statements), (2) perceived benefits of diphtheria vaccination (6 statements), (3) perceived barrier on diphtheria vaccination (4 statements), and (4) cues to action to undergo diphtheria vaccination (5 statements). The responses provided by the subjects were measured using a 5-point Likert scale: 1—strongly disagree, 2—disagree, 3—neutral, 4—agree, 5—strongly agree. The statements might be of positive or negative tones. For scoring of the statements, we took the given rating as the score for positively-toned statements; we subtract 6 from the rating given to obtain a score for negatively-toned statements. Possible scores ranged from 5 to 95. Higher perception score indicated more positive perception towards diphtheria vaccination (Pamungkas et al., 2019). Perceptions were previously shown to be stable (Benyamini and Friedman, 2011; Castro et al., 2012), hence it was assumed that perceptions measured in June 2019 could reflect the perception towards vaccination before clinical rotations, which were 6–18 months before the questionnaire was administered (Pamungkas et al., 2019).

The practice part of the questionnaire consisted of 7 questions that assessed intention to self-vaccinate and to promote vaccination to others. The subjects responded with a yes/no answer. A score of 1 is given for each positive answer, 0 for each negative answer, to obtain a total score between 0 and 7. The higher the score, the greater the intention to self-vaccinate and to promote vaccination to others (Whittaker, 2018).

### 2.3 Diphtheria toxin antibody measurement

Quantitative determination of IgG-class antibody against *Corynebacterium diphtheria* toxin was done based on enzyme-linked immunosorbent assay (ELISA) technique using commercial test kits (Diphtheria Toxin IgG ELISA; IBL International, Hamburg, Germany) following the manufacturer's instructions. The measurement was done by PT. BioFarma, Bandung, Indonesia (Syafriati et al., 2020; International, 2012).

Diphtheria toxin antibody was expressed in international unit/milliliter (IU/mL). Subjects were divided into those with reliable protection against diphtheria and those without. Subjects with diphtheria toxin IgG level ≥0,10 IU/mL were considered to have reliable protection against diphtheria (International, 2012).

### 2.4 Data analysis

Data analysis was performed using GraphPad Prism version 7.0 (GraphPad Software, California, United States of America). All data were tested for normal distribution, and comparisons were made with parametric tests for normally distributed variables and non-parametric tests for non-normally distributed variables. The point-biserial correlation test was performed for the correlation analysis.

### 2.5 Ethical aspects

The study protocol was approved by the Health Research Ethical Committee of Hasan Sadikin Hospital numbered LB.02.01/X.6.5/266/2020.

## 3 Results

### 3.1 Study participants

Twenty-eight subjects satisfied the inclusion and exclusion criteria. Table 1 showed the baseline characteristics of our subjects. Subjects were of similar age, and most (71,4%) were female. Subjects were not significantly different between those with and without reliable protection against diphtheria, except for the antibody titer after Td booster. Knowledge about diphtheria and diphtheria vaccination was not significantly different between the two groups. None of the baseline characteristics had significant association with perception score.

**Table 1:** Baseline characteristics.

Characteristics	All subjects (n ​= ​28)	Subjects without reliable protection (n ​= ​5)	Subjects with reliable protection (n ​= ​23)	p-value
Female sex, n (%)	20 (71,4)	4 (80,0)	16 (69,6)	0,646
Age, years, median (range)	22 (20–24)	22 (20–23)	22 (20–24)	0,871
Time elapsed since last Td booster^a^, months, median (range)	18 (6–18)	18 (6–18)	18 (6–18)	0,755
Previous negative experience related to vaccinations, n(%)	4 (14,3)	2 (40,0)	2 (8,7)	0,075
Knowledge score, median (range)	8 (4–10)	7 (5–10)	8 (4–10)	0,878
Diphtheria antitoxin levels after Td booster, IU/mL, median (range)∗	0,28 (0,01–3,47)	0,07 (0,01–0,09)	0,50 (0,10–3,47)	0,001

a Td vaccination prior to clinical rotation; Td: tetanus-diphtheria; IU/mL: international unit per milliliter; ∗p ​< ​0,05 with Mann-Whitney test.

### 3.2 Perception

Table 2 showed subjects’ knowledge and perception towards diphtheria vaccination among subjects with reliable protection against diphtheria and without. Study subjects had similar knowledge on diphtheria and its vaccination. Those with reliable protection levels of diphtheria toxin antibody had more positive perception compared to those without reliable protection (perception score 80,6 ​± ​5,4 vs 69,0 ​± ​1,8, *p* ​= ​0,0001). Subjects with reliable protection against diphtheria also perceived themselves to have less barrier for undergoing vaccination (perceived barrier 15,6 ​± ​2,1 vs 13,0 ​± ​1,8, *p* ​= ​0,0083) compared to those without reliable protection. All subjects seemed to perceive diphtheria as threatening, perceived diphtheria booster vaccination as beneficial and that external cues to action were supportive towards vaccination.

**Table 2:** Knowledge and perception score of subjects based on antibody levels after Td booster.

Characteristics	Diphtheria antitoxin levels		p-value
	Without reliable protection (n ​= ​5)	With reliable protection (n ​= ​23)	
Perception score, mean ​± ​SD	69,0 ​± ​1,8	80,6 ​± ​5,4	0,0001^a^∗
Perceived threat, median (IQR)	18,0 (14,3–18,8)	18,5 (18,0–20,0)	0,0790^b^
Perceived benefit, median (IQR)	22,5 (21,3–26,8)	28,0 (25,3–29,0)	0,1404^b^
Perceived barrier, mean ​± ​s.d.	13,0 ​± ​1,8	15,6 ​± ​2,1	0,0083^a^∗
Cue to action, mean ​± ​s.d	15,5 ​± ​3,1	19,8 ​± ​3,4	0,0810^a^

∗p ​< ​0,05.

a Calculated using T-test.

b Calculated using Mann-Whitney test.

**Fig. 1:**
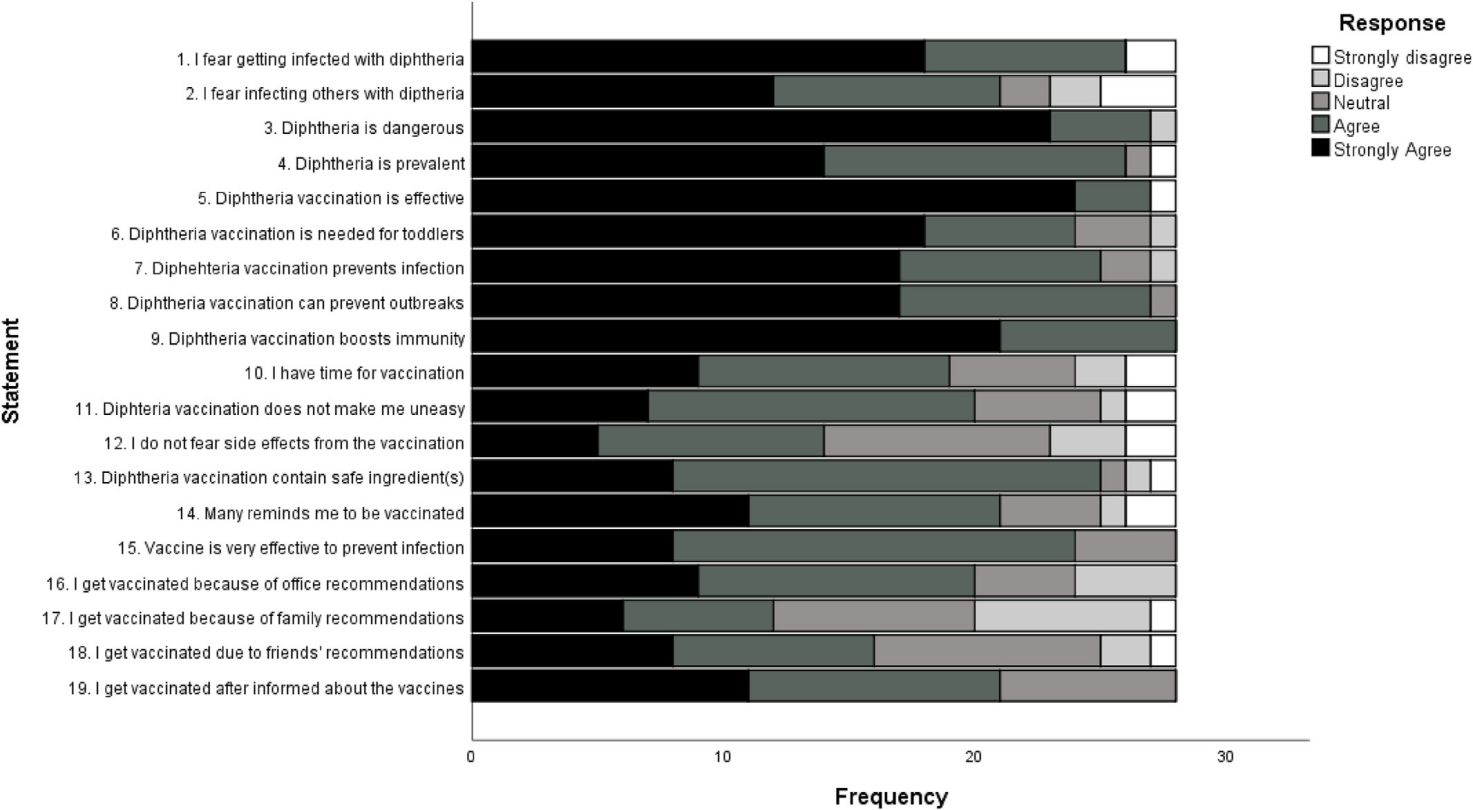
Responses of statements.

This could further be seen more clearly in responses for each perception statement shown in Fig. 1. Statement 1–4 assessed subjects' perceived threat of diphtheria. Most subjects agreed or strongly agreed that diphtheria was a threat: that the disease might infect them and might be severe. Statement 5–9 and 15 evaluated subjects' perceived benefits of vaccination. Most subjects agreed or strongly agreed that diphtheria vaccination was effective. Statement 10–14 and 16–19 respectively considered subjects' perceived barrier on getting vaccinated and their perception of external cues that encouraged vaccination. Here, most subjects chose ‘agree’ and ‘strongly disagree’ that some barriers for undergoing vaccination were low: they felt they had enough time to get vaccinated, that the vaccine was safe, and that their surroundings supported vaccination. However, more subjects chose ‘neutral’ for statements that assessed perceived barriers and perceived cues to action compared to those which assessed perceived threat and benefits. This indicated that there might be some hesitancy that needed to be addressed.

### 3.3 Diphtheria toxin antibody level

The median value of diphtheria toxin IgG antibody level was 0,28 (range 0,09–3,47) IU/mL. Twenty-three (82,1%) subjects had reliable protection level of antibody, which was ≥0,10 IU/mL.

### 3.4 Correlation between perception and diphtheria toxin antibody level

**Fig. 2:**
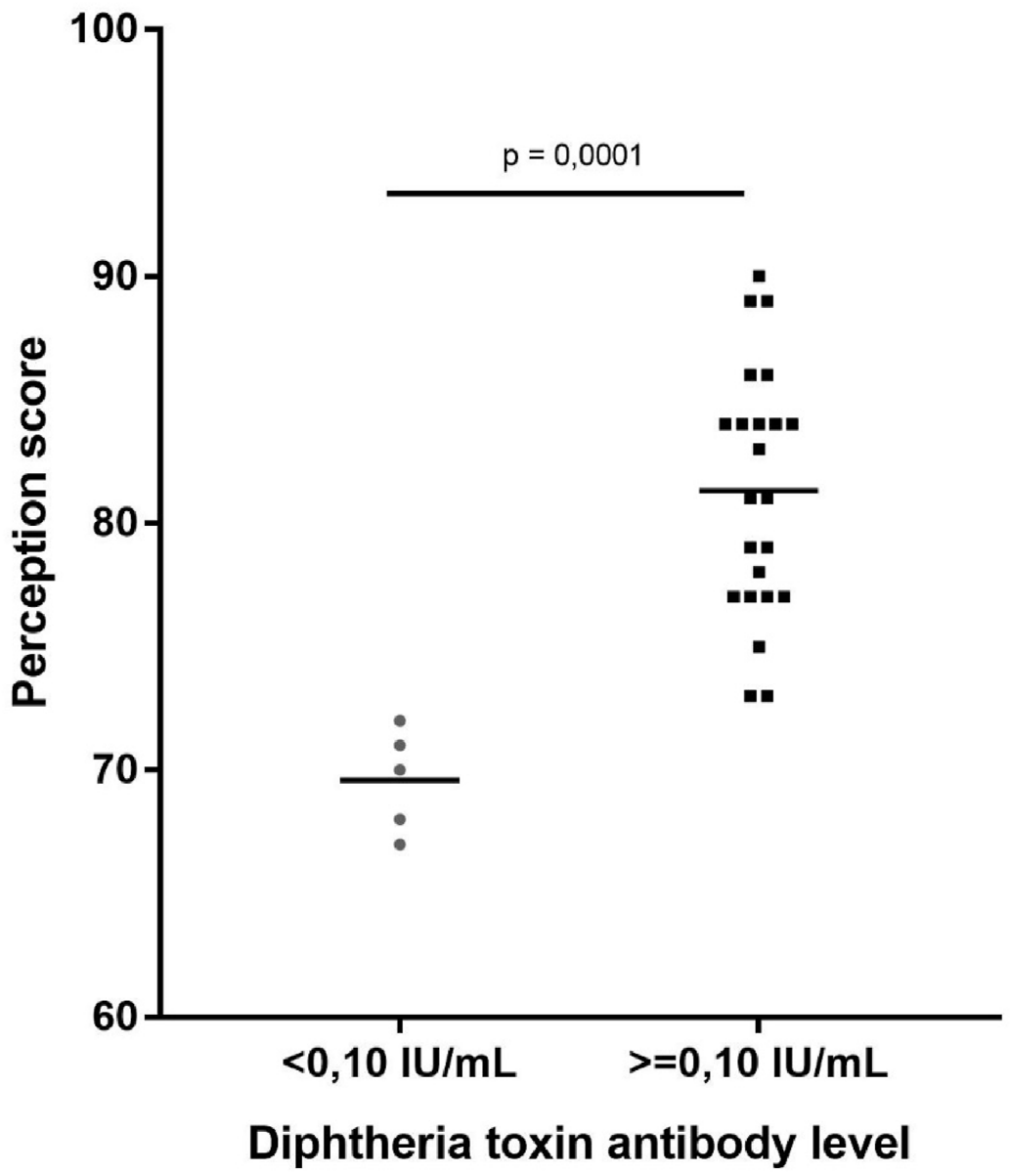
Scatterplot of perception score vs diphtheria antitoxin levels a year after Td booster.

Scatterplot between presence of reliable protection against perception score is shown in Fig. 2. Perception score showed strong, positive correlation to presence of reliable protection against diphtheria (R ​= ​0,705, p ​< ​0,001).

**Fig. 3:**
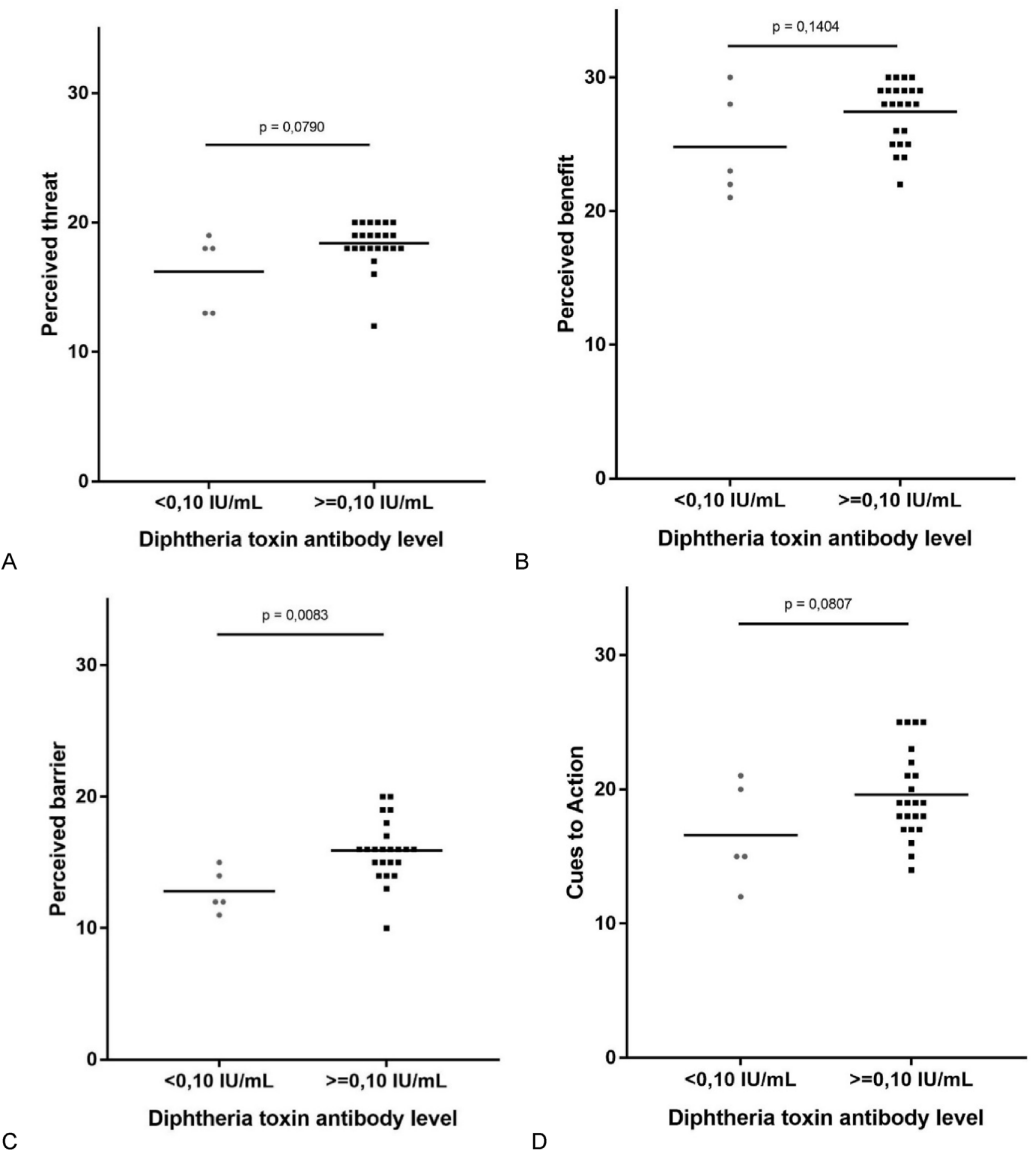
Scatterplot of (A) perceived threat, (B) perceived benefits, (C) perceived barrier, (D) cues to action to diphtheria vaccination vs diphtheria toxin antibody titer a year after Td booster.

Scatterplots of the presence of reliable protection against components of each perception are shown in Fig. 3. Components of the perception score which showed positive correlation to presence of reliable protection against diphtheria was perceived barrier (R ​= ​0,489, p ​= ​0,008) and perceived threat (R ​= ​0,402, p ​= ​0,034). Perceived benefits and cues to action did not show significant correlation to presence of reliable protection.

## 4 Discussion

This study was conducted to determine correlation between perception towards diphtheria vaccination and presence of protective antibody against diphtheria. It was found that there was strong positive correlation between perception towards diphtheria vaccination and presence of protective antibody against diphtheria.

Illness perception was previously shown to be associated with stress level in many illnesses such as gastrointestinal cancers, chronic lymphocytic leukemia, and atrial fibrillation (Westbrook et al., 2016; Miceli et al., 2019; McCabe and Barnason, 2012). Illness perception had cognitive and emotional components (Hagger et al., 2017). Emotional aspect of perception had been shown to affect stress level, fatigue, and depression in chronic lymphocytic leukemia (Westbrook et al., 2016). Subjects with and without reliable antibody protection against diphtheria in this study had similar knowledge on diphtheria and diphtheria vaccination, hence it was more likely that the emotional component was more dominant in determining whether the subject had positive or negative perception on vaccination.

Psychoneuroimmunology studies showed that chronic and acute stress influenced antibody response after Hepatitis B and influenza vaccinations (Glaser et al., 1992; Kiecolt-Glaser et al., 1996). It had also been shown that individuals with positive affect had higher antibody response after vaccination (Marsland et al., 2006; Jenkins et al., 2018). Influence of psychosocial factors, such as stress, on diphtheria vaccine response had not been studied (Zimmermann and Curtis, 2019). This study was one of the first to suggest that perception also influenced antibody response after diphtheria vaccination. Those with more positive perceptions towards diphtheria vaccination were more likely to obtain protective antibody.

Illness perception had been shown to be relatively stable (Benyamini and Friedman, 2011; Castro et al., 2012), therefore, we assumed that the measurement of perceptions in June 2019 reflected the perceptions of subjects during vaccination prior to their clinical rotations. De Castro et al. showed that breast cancer patients in Brazil had similar illness perception at baseline and after one year follow up (Castro et al., 2012). Few internal and external factors might influence illness perception, for example age, gender, personality, and culture (Benyamini and Friedman, 2011). The subjects in this study had homogenous age and came from relatively similar culture, hence these factors were probably not confounding the measurement of perception in this study.

Several factors are known to affect diphtheria antibody levels after vaccination, namely age, time since previous diphtheria vaccination, comorbidities (chronic kidney disease and liver failure), and genetic polymorphisms (Zimmermann and Curtis, 2019).

Indonesia started their National Immunization Program for infants and children in 1977, of which diphtheria vaccination was one of the basic immunization obliged for all infants and children (Indonesian Society of Internal Medicine, 2017). Elderly people were born before this program was available, hence they might not have completed basic immunization during their childhood and therefore had less antibody compared to the younger population. However, the subjects in this study were homogenous in terms of age as they were all medical interns undergoing similar stage of education, therefore age was probably not a confounding in the measurement of diphtheria toxin antibody level.

Booster for Td vaccination was recommended every 10 years in most countries such as United States, Canada, and Indonesia (Indonesian Society of Internal Medicine, 2017; Center of Disease Control and Prevention United States of America, 2021; Canada Government. Recomm, 2020). Recent study showed that diphtheria toxin antibody level declined with an estimated half-life of 18–51 years while tetanus toxin antibody had an estimated half-life over 11–17 years (Hammarlund et al., 2016). Subjects in this study had their diphtheria vaccination 6–18 months before antibody measurement was taken. No data of the subjects’ previous immunizations were available due to the secondary data nature of this study. Indonesian Government scheduled diphtheria, tetanus and pertussis vaccination for infants and Td booster for elementary school students aged 6–12 years since 1977 (Indonesian Society of Internal Medicine, 2017). Since the subjects were born after 1977, it was thought that there was homogenous exposure to previous Td vaccinations.

None of the subjects had chronic kidney disease nor liver failure. Patients with chronic kidney disease have poorer responses to diphtheria vaccination compared to healthy subjects due to toxic effects of uremia on immunity (Krüger et al., 2001; Kato et al., 2008). Those with chronic liver disease have a faster decline in diphtheria antibody level (Balloni et al., 1999).

This study did not consider genetic polymorphisms possible factor which might influence the antibody level of subjects. Single nucleotide polymorphism in IL-10 gene was shown to be responsible for 49% of the variation in diphtheria toxin antibody level after childhood immunization (Newport et al., 2004). Various studies shown that polymorphism in IL-10 gene existed in Indonesian population (Andalas et al., 2016). Syafriati et al. showed that the subjects in this study achieved protective antibody levels 1 month after given Td vaccinations (Syafriati et al., 2020), hence the subjects’ immune system appeared to respond well to the vaccination.

This study revealed the need to have positive outlooks on vaccination to achieve protective antibodies after vaccination. The subjects in this study were medical interns who were used to observe diphtheria cases and were more knowledgeable about diphtheria vaccination compared to the general public. These knowledge and experiences tended to make medical students more likely perceive diphtheria as a preventable but potentially fatal disease compared to the general public. These translated into good perceived threats about diphtheria as well as good perceived benefits of diphtheria vaccination in this study. A lot of medical interns in this study still feared vaccine side effects. Some viewed vaccines to contain potentially harmful elements. These might contribute to vaccine hesitancy among medical interns. Tying in to Leventhal's Common-Sense Model of Self-Regulation, the subjects in this study might be more homogenous in terms of their cognitive representation of the perception towards vaccination compared to the general population. It was possible that the differences in perception among our subjects was due to the emotional representation of perception.

As our data showed that even health care workers such as medical interns had reservations about vaccinations, it was necessary to educate people on these barriers, such as knowledge on side effects, religious issues about vaccination, as those can lead to negativity about vaccinations and may hinder the attainment of protective antibody. Health providers and administrators should involve religious leaders to educate about the safety of vaccines. Strong protocols on adverse effects following immunizations (AEFI) should be implemented to keep people's good faith in the vaccine delivery system. These measures might contribute in shaping more positive perceptions on vaccination, which was shown to associate with protective antibody level.

Finally, this study was not without limitations. Secondary data from previously unpublished research was used, hence certain information, such as prior vaccination history or records, was not available. Analysis of those data might give a better interpretation of the immunity obtained by the subjects after Td vaccination. Furthermore, it was not possible to longitudinally measure perception because the original studies were conducted using cross-sectional method. Instead, we assumed that perceptions were relatively stable over time. Lastly, measurement of stress and its biomarkers would provide more valuable evidence on the correlation of perception and antibody response. Despite these limitations, this study might still be useful in providing insights on how to improve antibody response after vaccination by increasing positive perception.

## 5 Conclusion

In conclusion, perception on diphtheria vaccination is strongly correlated to protective antibody among medical interns. Health care workers need to understand the benefits of positive perception toward vaccination and its association with antibody response after vaccination to encounter vaccine hesitancy, because health care workers have an important role as educator of patients and their families. Health care workers also need to be aware of emotional aspects of perception while educating about vaccines. Collaboration between health providers and administrators, community leaders as well as religious heads is needed. This is especially paramount in this time of Coronavirus Disease-19 (COVID-19) pandemic, in which vaccination program is one of the key prevention measures to overcome the disease. This study suggests that there are possible roles of psychological factors, such as perception towards COVID-19 vaccination and COVID-19-related stress, on vaccination responses. It is also raised that the emotional aspect of the pandemic might influence the outcome of the vaccination efforts as well as the disease. These psychological areas are worth exploring in further studies.

## Fundings

This study received no external funding. The studies from which we obtained secondary data for this study were funded by by Hibah Internal Universitas Padjadjaran year 2018 and supported by PT. Bio Farma.

## Declaration of competing interest

The authors declare that they have no known competing financial interests or personal relationships that could have appeared to influence the work reported in this paper.

## References

[bib1] Adjei D.N., Stronks K., Adu D., Beune E., Meeks K., Smeeth L. (2019). Cross-sectional study of association between psychosocial stressors with chronic kidney disease among migrant and non-migrant Ghanaians living in Europe and Ghana: the rodam study. BMJ Open.

[bib2] Agency HDaR. (2019).

[bib3] Andalas M., Hakimi M., Nurdiati D.S., Astuti I., Ichsan I., Wahyuniati N. (2016). Lack of association between the –1082 (a/G) il-10 polymorphism (Rs1800896) and spontaneous preterm birth in the Indonesian acehnese population. Polish Annals of Medicine.

[bib4] Balloni A., Assael B.M., Ghio L., Pedrazzi C., Nebbia G., Gridelli B. (1999). Immunity to poliomyelitis, diphtheria and tetanus in pediatric patients before and after renal or liver transplantation. Vaccine.

[bib5] Benyamini Y., Friedman H.S. (2011). The Oxford Handbook of Health Psychology.

[bib6] Canada Government (2020). Recommended immunization schedules: Canadian immunization guide. https://www.canada.ca/en/public-health/services/publications/healthy-living/canadian-immunization-guide-part-1-key-immunization-information/page-13-recommended-immunization-schedules.html.

[bib7] Castro E.K., Kreling M., Ponciano C., Meneghetti B.M., ChemI C.M. (2012). Longitudinal assessment of illness perceptions in young adults with cancer. Psicol. Reflexão Crítica.

[bib8] Center of Disease Control and Prevention United States of America (2021). Immunization schedules. https://www.cdc.gov/vaccines/schedules/hcp/imz/adult.html.

[bib9] Glaser R., Kiecolt-Glaser J., Bonneau R.H., Malarkey W., Kennedy S., Hughes J. (1992). Stress-induced modulation of the immune response to recombinant hepatitis B vaccine. Psychosom. Med..

[bib10] Grebe K.M., Takeda K., Hickman H.D., Bailey A.L., Embry A.C., Bennink J.R. (2010). Cutting edge: sympathetic nervous system increases proinflammatory cytokines and exacerbates influenza a virus pathogenesis. J. Immunol..

[bib11] Grisanti L.A., Woster A.P., Dahlman J., Sauter E.R., Combs C.K., Porter J.E. (2011). Α1-Adrenergic receptors positively regulate toll-like receptor cytokine production from human monocytes and macrophages. J. Pharmacol. Exp. Therapeut..

[bib12] Hagger M.S., Koch S., Chatzisarantis N.L.D., Orbell S. (2017). The Common Sense model of self-regulation: meta analysis and test of a process model. Psychol. Bull..

[bib13] Hammarlund E., Thomas A., Poore E.A., Amanna I.J., Rynko A.E., Mori M. (2016). Durability of vaccine-induced immunity against tetanusand diphtheria toxins: a cross-sectional analysis. Clin. Infect. Dis..

[bib14] Harapan H., Alleta A., Anwar S., Setiawan A.M., Maulana R., Wahyuniati N. (2018). Attitudes towards zika virus infection among medical doctors in aceh province, Indonesia. J Infect Pub Health.

[bib15] Harjaningrum A.T., Kartasasmita C., Orne-Gliemann J., Jutand M.-A., Koeck N.G.J.-L. (2013). A qualitative study on knowledge, perceptions, and attitudes of mothers and health care providers toward pneumococcal conjugate vaccine in Bandung, West Java, Indonesia. Vaccine.

[bib16] Djauzi S., Rengganis I., Sundoro J., Koesnoe S., Soegiarto G., Maria S., Indonesian Society of Internal Medicine (2017). Immunization Guide in Adults 2017. 4.

[bib17] International I.B.L. (2012). GMBH II.

[bib18] Jenkins B.N., Hunter J.F., Cross M.P., Acevedo A.M., Pressman S.D. (2018). When is affect variability bad for health? The association between affect variability and immune response to the influenza vaccination. J. Psychosom. Res..

[bib19] Kato S., Chmielewski M., Honda H., Pecoits-Filho R., Matsuo S., Yuzawa Y. (2008). Aspects of immune dysfunction in end-stage renal disease. Clin. J. Am. Soc. Nephrol..

[bib20] Kiecolt-Glaser J.K., Glaser R., Gravenstein S., Malarkey W.B., Sheridan J. (1996). Chronic stress alters the immune response to influenza virus vaccine in older adults. Proc. Natl. Acad. Sci. U. S. A..

[bib21] Krüger S., Müller-Steinhardt M., Kirchner H., Kreft B. (2001). A 5-year follow-up on antibody response after diphtheria and tetanus vaccination in hemodialysis patients. Am. J. Kidney Dis..

[bib22] Leventhal H., Phillips L.A., Burns E. (2016). The common-sense model of self-regulation (csm): a dynamic framework for understanding illness self-management. J. Behav. Med..

[bib23] Louveau A., Smirnov I., Keyes T.J., Eccles J.D., Rouhani S.J., Peske J.D. (2015). Structural and functional features of central nervous system lymphatic vessels. Nature.

[bib24] Lu X., Juon H.-S., He X., Dallal C.M., Wang M.Q., Lee S. (2019). The association between perceived stress and hypertension among Asian Americans: does social support and social network make a difference?. J. Community Health.

[bib25] Marsland A.L., Cohen S., Rabin B.S., Manuck S.B. (2006). Trait positive affect and antibody response to hepatitis B vaccination. Brain Behav. Immun..

[bib26] McCabe P.J., Barnason S.A. (2012). Illness perceptions, coping strategies, and symptoms contribute to psychological distress in patients with recurrent symptomatic atrial fibrillation. J. Cardiovasc. Nurs..

[bib27] Miceli J., Geller D., Tsung A., Hecht C.L., Wang Y., Pathak R. (2019). Illness perceptions and perceived stress in patients with advanced gastrointestinal cancer psychooncology.

[bib28] Miller G.E., Cohen S., Ritchey A.K. (2002). Chronic psychological stress and the regulation of pro-inflammatory cytokines: a glucocorticoid-resistance. Model Health Psychol.

[bib29] Newport M.J., Goetghebuer T., Weiss H.A., Group T.M.G.T.S., Whittle H., Siegrist C.A. (2004). Genetic regulation of immune responses to vaccines in early life. Gene Immun..

[bib30] Pamungkas T.C., Pranggono E.H., Wisaksana R. (2019).

[bib31] Pronyk P., Sugihantono A., Sitohang V., Moran T., Kadandale S., Muller S. (2019). Vaccine hesitancy in Indonesia. Lancet Planetary Health [Internet].

[bib32] Slavich G.M. (2019). The Oxford Handbook of Stress and Mental Health.

[bib33] Sorrells S.F., Caso J.R., Munhoz C.D., Sapolsky R.M. (2009). The stressed cns: when glucocorticoids aggravate inflammation. Neuron.

[bib34] Syafriati E., Pranggono E.H., Wachjudi R.G. (2020). Diphteria Toxin Antibody Titer before and after Tetanus-Diphtheria Vaccination and Adverse Events Following Immunizations in Adults. 33rd World Congress on Vaccines and Immunization.

[bib35] Syiroj A.T.R., Pardosi J.F., Heywood A.E. (2019). Exploring parents' reasons for incomplete childhood immunisation in Indonesia. Vaccine.

[bib36] Tosepu R., Gunawan J., Effendy D.S., Ahmad L.O.A.I., Farza A. (2018). The outbreak of diphtheria in Indonesia. Pan Afr J Med.

[bib37] Westbrook T.D., Maddocks K., Andersen B.L. (2016). The relation of illness perceptions to stress, depression, and fatigue in patients with chronic lymphocytic leukaemia. Psychol. Health.

[bib38] Whittaker A.C. (2018). The vaccination model in Psychoneuroimmunology research: a review. Methods Mol. Biol..

[bib39] Yufika A., Wagner A.L., Nawawi Y., Wahyuniati N., Anwar S., Yusri F. (2020). Parents' hesitancy towards vaccination in Indonesia: a cross-sectional study in Indonesia. Vaccine.

[bib40] Zimmermann P., Curtis N. (2019). Factors that influence the immune response to vaccination. Clin Microbiol Rev [Internet].

